# Global Status, Recent Trends, and Knowledge Mapping of Olive Bioactivity Research Through Bibliometric Analysis (2000–2024)

**DOI:** 10.3390/foods14081349

**Published:** 2025-04-14

**Authors:** Manuel Garrido-Romero, Marina Díez-Municio, F. Javier Moreno

**Affiliations:** 1 Instituto de Investigación en Ciencias de la Alimentación, CIAL (CSIC-UAM), Nicolás Cabrera 9, 28049 Madrid, Spain; manuel.garrido@pharmactive.eu; 2 Pharmactive Biotech Products SLU, Faraday 7, 28049 Madrid, Spain; mdiez@pharmactive.eu

**Keywords:** olive, bioactive compounds, nutraceuticals, Mediterranean diet, oil, functional foods

## Abstract

Over the past two decades, both academic and industrial interest in olive bioactive compounds has grown significantly due to their remarkable health benefits, such as antioxidant, anti-inflammatory, and cardioprotective properties. These compounds, found in both olive fruit and leaves, have become a central focus in the research on functional foods and nutraceuticals. A comprehensive bibliometric analysis of scientific publications from 2000 to 2024 highlights a notable increase in this field, with 2228 documents published in high-impact journals with an estimated annual growth rate of 0.2694 year^−1^, particularly in the last decade. This surge reflects the growing recognition of olive bioactive compounds’ potential in promoting human health through nutritional and therapeutic interventions, and their role in the expanding nutraceutical industry. This growth is further reaffirmed by patent analysis, which shows a significant rise in industrial interest and patent filings related to olive bioactive compounds. The analysis also examined nearly 6000 keywords to identify the most influential research domains, pinpoint knowledge gaps, and reveal the most important bioactive compounds in olives and their potential in preventing various human diseases.

## 1. Introduction

In recent years, both consumers and public health professionals have increasingly recognized the critical role of diet in disease prevention. Consequently, interest in natural bioactive compounds, derived from plants, fungi, bacteria, animals and agri-food industrial residues, has surged. These compounds offer promising antioxidant [1], anti-inflammatory [2], antimicrobial [3], and antiproliferative properties [4], along with significant immunomodulatory and cardioprotective benefits [5]. Nutraceuticals, defined as food or food components that provide beneficial health effects, have emerged as attractive tools for enhancing well-being. While not substitutes for pharmaceutical drugs, they have demonstrated efficacy in both preventing and alleviating various conditions [6].

Over the past few years, numerous scientific reports have underscored the nutraceutical and nutritional significance of the Mediterranean diet, and these findings have suggested that incorporating this dietary pattern contributes to a decreased incidence of pathologies associated with oxidative stress and inflammation, including cardiovascular diseases and cancer [7]. One of the main components of this diet is olive *(Olea europaea*), which, alongside cereals, is one of the most extensively cultivated crops in the Mediterranean area, with over 10 million hectares of olive groves worldwide—95% of them located in the Mediterranean basin [8]. Its cultivation has played a crucial role in the economic and dietary traditions of Mediterranean civilizations, spreading over time to countries such as Spain, Italy, France, Greece, and Turkey [9]. Beyond its economic relevance, the olive is also valued for its rich content of bioactive compounds with well-documented health benefits. Its derivatives, such as extra-virgin olive oil and olive leaves, are particularly abundant in antioxidants, including oleuropein, oleic acid, and hydroxytyrosol, which play a key role in protecting against oxidative stress and chronic diseases [10].

To the best of our knowledge, the previous bibliometric studies on olives have taken a broad approach, without specifically addressing their bioactive compounds and health benefits. For example, they have focused on the olive oil supply chain [11], global research about olive oil [12], or the traceability of olive oil’s origins [13]. In light of this, this study aims to bridge that gap by investigating the bioactivity of various olive-derived compounds, aligning with the growing need to develop health-promoting products and repurpose agro-industrial by-products to reduce the carbon footprint. Hence, we have gathered and curated data from a wide number of publications on the bioactive compounds of olive and its derivatives, covering the period from 2000 to 2024. The main objective of this bibliometric analysis is to answer the following research questions, which we consider of interest to researchers and manufacturers in this field:

(a) What are the global trends in scientific production on the bioactivity of the compounds found in the olive?
(b) To what extent is technology being transferred from academia to the food industry?
(c) What are the major research topics in this field?
(d) How has this field evolved over time?
(e) Which are the main knowledge gaps and challenges to overcome?

## 2. Material and Methods

### 2.1. Data Collection

The data analyzed in this paper were obtained from the Scopus database and Clarivate Analytics’ Web of Science, both of which are widely used in bibliometric studies. An exhaustive search was performed on Scopus using the query string: TITLE-ABS-KEY (“olive” OR “olea” AND “bioactive” OR “bioactivity”), restricting the publication date from January 2000 to December 2024. Patent data were retrieved from the Espacenet database using the query (nftxt = “olive” OR nftxt = “olea”) AND (nftxt = “bioactive” OR nftxt = “bioactivity”), also limited to documents published between 2000 and 2024.

### 2.2. Analysis of Keywords

In this analysis, we considered both the keywords provided by the authors and those indexed by the Scopus search engine. A total of 5595 keywords were retrieved from the search results. To streamline the dataset and ensure relevance, a minimum occurrence threshold of 15 repetitions across the papers was applied, resulting in 72 keywords that met this criterion. In addition, a selection process was applied to avoid redundancy: when two terms had similar meanings, the term with the highest frequency of occurrences was chosen. For example, between “anti-inflammatory” and “anti-inflammatory activity,” the former was selected as it appeared 17 times, compared to 10 occurrences of the latter.

To further refine the dataset, irrelevant or overly broad terms, such as “optimization” or “chemometrics”, which did not contribute directly to the scope of our analysis, were excluded. This ensured that the final set of keywords accurately represented the central themes of the research. The resulting keywords were then used to identify key research trends, themes, and areas of focus within the literature on olive bioactivity.

VOSviewer software (version 1.6.20 available online at www.vosviewer.com (accessed on 10 January 2025)) was used for clustering and visualizing keyword relationships. One important parameter is link strength, which indicates the strength of co-occurrence between keywords. This is represented by the thickness of the lines connecting the nodes; thicker lines mean stronger relationships between the keywords. Another key feature is the node size, which reflects the frequency of a keyword in the dataset—larger nodes show more common terms [14].

VOSviewer also allows the identification of clusters, where related keywords are grouped together. The proximity of nodes within a cluster suggests a stronger association between the topics. Additionally, the density visualization feature highlights areas of high research activity, showing concentrations of keywords that are more frequently studied. These parameters helped in mapping the evolving trends and connections within olive bioactivity research, providing deeper insights into the key research themes.

## 3. Progression of Scientific Production

**Figure 1:**
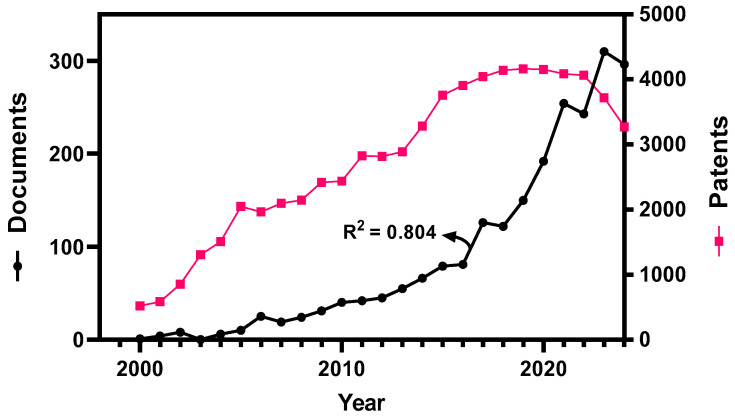
Trend in the number of publications and patents per year for bioactive compounds in olive in the period 2000–2024.

Figure 1 shows the evolution in the number of scientific documents related to the bioactivity of olive (*Olea europaea*) over the past twenty-five years, reaching a total of 2228 documents. This evolution can be fitted to an R^2^ value of 0.804 and a growth rate of 0.2694 year^−1^, which is higher than that corresponding to the overall number of scientific publications in this field [15]. Thus, there is a growing interest in this subject due to the emerging need to obtain bioactive compounds from plant-based samples that can improve human health.

This trend is also confirmed by the study of the patents obtained in this matter. The transference of knowledge from academia to the nutraceutical and food industry is unquestionably relevant as the number of patents has increased ten times in the last two decades (Figure 1), highlighting the fact that the market is growing for food-derived products that can improve the health of the consumer.

However, since 2019, there has been a marked decline in the number of patents filed in this field. A plausible explanation for this downturn is the market’s maturation, with many of the most promising bioactive compounds from olive-derived products already patented and in various stages of commercialization. This maturation phase has prompted companies to focus on optimizing the bioavailability and functionality of these compounds in new products. Furthermore, increasing regulatory scrutiny and more stringent approval processes for nutraceuticals and functional foods may have contributed to the slower pace of innovation. As the industry becomes more saturated, companies may be redirecting efforts toward refining existing formulations rather than pursuing new patents, focusing on enhancing the effectiveness and marketability of already established products.

## 4. Keyword Analysis

**Figure 2:**
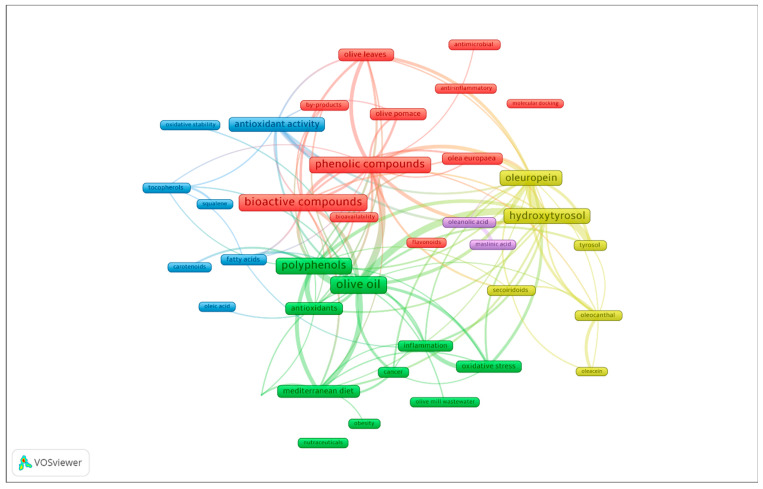
Clusters of keywords in publications on the bioactive compounds found in olive through the period 2000–2024.

The 38 keywords selected for the analysis of the keywords were added to the software VOSviewer, and the results are shown in Figure 2, which illustrates the distribution of keywords according to their co-occurrence in scientific articles. Five clusters or keyword communities were identified, each represented by different colors. The size of the label was determined based on their frequency of occurrence in the literature. Furthermore, the analysis reveals distinct patterns in the relationships between keywords, highlighting the key areas of focus in the research field. In the following sections, each of the clusters will be explained in detail, alongside an exploration of the relationships between the members within each cluster, offering insights into the prevailing research trends and their interconnections.

### 4.1. Bioactive Compounds in Olives: Health Benefits and By-Product Valorization (Red Cluster)

This is the largest group, comprising 12 items, with the most prominent keywords being bioactive (189 occurrences) and phenolic compounds (181), underscoring the central focus of research on these molecules. Bioactive compounds—such as phenolic compounds (e.g., flavonoids), alkaloids, terpenes and carotenoids—are naturally occurring in plants, animals, and microorganisms, attracting extensive research due to their diverse health benefits. Known for their antimicrobial (35), anti-inflammatory (23), antioxidant, and anticarcinogenic properties, they also support cardiovascular health, metabolic regulation, neuroprotection, and immune modulation [16,17]. Additionally, they influence gut microbiota and cellular signaling pathways, making them promising candidates for functional foods, pharmaceuticals, and nutraceuticals [18]. The growing demand for natural, health-promoting products continues to drive research into their mechanisms, bioavailability, and applications. Advances in food science, biotechnology, and pharmacology are uncovering new sources and innovative ways to integrate these compounds into dietary and therapeutic strategies for improved human health.

Among the various classes of bioactive compounds, flavonoids (31) stand out due to their potent antioxidant properties and wide-ranging health benefits [19], as they are highly effective at reducing free radical formation and scavenging free radicals [20]. In addition to their strong antioxidant activity, flavonoids also exhibit anti-inflammatory effects, the ability to prevent mutations, and inhibition of carcinogenesis [21]. Furthermore, they are able to regulate key cellular enzyme functions, contributing to their therapeutic and health-promoting properties [19]. The specific types of flavonoids found in olives depend on the part of the olive being studied, with the most common flavonoids identified including flavonols (such as quercetin and kaempferol), flavones (such as luteolin), anthocyanins (such as diosmetin), and other compounds (such as taxifolin and tiliroside) [22,23,24]. To fully understand how these compounds provide health benefits, it is essential to precisely identify the specific flavonoids present in olives and then investigate the exact mechanisms through which they exert their protective effects in combating diseases.

With the growing demand for flavonoids and other bioactive compounds, researchers are increasingly exploring sustainable and cost-efficient sources. Notably, agricultural by-products (35), often dismissed as mere industrial waste, have emerged as valuable repositories of these bioactive molecules. The utilization of these underexploited resources not only facilitates the extraction of high-value compounds but also aligns with sustainable and environmentally responsible practices. To propel this approach forward, novel characterization techniques within the olive oil industry are being developed to promote a circular economy [25,26] as will be discussed below. Among the most extensively researched olive by-products are olive leaves (79) and olive pomace (58):

Olive leaves have long been utilized in traditional remedies across European and Mediterranean regions, owing to their rich content of bioactive compounds, particularly phenolic compounds, which are renowned for their health-promoting properties [27]. Among the various methods employed for polyphenol extraction from olive leaves, natural deep eutectic solvents (NADES) are emerging as promising, sustainable alternatives, driven by growing ecological and toxicological concerns [28]. The application of NADES in olive leaf extraction has yielded promising results, particularly in extracting bioactive compounds such as hydroxytyrosol [29], oleuropein, rutin [30], and caffeic acid [31]. However, further research is needed to explore their full potential, as NADES, being green solvents, offer a sustainable and efficient alternative to traditional methods, enabling the extraction of bioactive compounds with minimal environmental impact.

Beyond their health benefits, the phenolic compounds from olive leaves are also being explored for their application in active packaging, owing to their antimicrobial and antioxidant properties, which could enhance food preservation by extending shelf life [32,33]. Given their antimicrobial properties, olive leaf extract has been studied as a natural preservative for various food products, including chilled poultry meat [34], non-thermally stabilized foods [35], and fresh hamburger [36]. Further investigation is warranted to assess its potential in other food categories, under diverse storage conditions, and in synergy with other natural preservatives. Moreover, exploring its efficacy across different packaging formats and its influence on sensory attributes such as taste, texture, and aroma would offer a more holistic understanding of its applicability as a preservative in the food industry.

Olive pomace, a significant by-product of olive oil production, serves as a valuable source of bioactive compounds with well-established health benefits [37]. Recent research has highlighted its potential as a rich reservoir of polyphenols, such as hydroxytyrosol and tyrosol, which can be successfully recovered using ultrasound-assisted extraction techniques [38]. Similarly, Atta et al. (2022) examined the hydro-ethanolic extract of olive pomace, which revealed high concentrations of phenolic compounds, including tannins and flavonoids, all possessing robust antioxidant properties [39].

In addition to its health benefits, olive pomace has been explored for its application in biodegradable materials for active packaging, demonstrating its capacity to enhance food stability and improve the quality and shelf-life of food products [40]. Antimicrobial assays have further confirmed the potent inhibitory effects of these enriched extracts against a range of harmful microorganisms (*E. coli*, *S. aureus*, and *N. sativa*, among others), strengthening their potential for use in food preservation and safety [41,42]. This underscores the multifaceted value of olive pomace, not only as a source of bioactive compounds but also as a promising candidate for sustainable food preservation solutions.

Furthermore, research is increasingly focused on fortifying olive oil with bioactive compounds derived from its by-products. For instance, studies have demonstrated that the antioxidant capacity and bioactive content of olive oil can be increased while maintaining acceptable quality parameters by enriching it with hydroxytyrosol derived from olive leaf extract [43]. Other methods involve incorporating phenolic compounds obtained from olive pomace and leaves through ultrasonic extraction [44] or derivatives of oleuropein [45]. Additionally, the application of these bioactive-rich extracts not only enhances the oil’s health-promoting properties but also provides an eco-friendly solution for reducing waste from olive oil production. This strategy paves the way for the creation of innovative products, such as enriched olive oils with superior functional properties, which could attract significant interest within the industry.

Finally, the last term of this cluster belongs to the technique of “molecular docking” (17), which is utilized to determine, in silico, how effectively a ligand molecule can bind to a specific macromolecule, such as an enzyme or a receptor [46], and is one of the techniques used in the last couple of years in this field. This approach represents an interesting avenue for future research, particularly in exploring the therapeutic potential of bioactive compounds. For instance, one study analyzed bioactive compounds from *Olea europaea* as potential inhibitors of the *Spike* protein and the main protease of SARS-CoV-2, identifying squalene as having the highest inhibition potential [47]. Molecular docking was also used to identify how bioactive compounds from *Olea europaea* interact with human tyrosinase, revealing oleuropein as the compound with the highest inhibitory capacity and binding affinity, suggesting its potential for skin health applications, such as regulating melanin production [48].

Additional studies have demonstrated that many phenolic compounds in olive interact with the active site of human pancreatic α-amylase, confirming their inhibitory effects and highlighting the antioxidant and anti-diabetic properties of these bioactive compounds [49]. Olive phenolics were also shown to inhibit some enzymes linked to depression, supporting their potential antidepressant effects associated with the Mediterranean diet [50]. These findings contribute to the growing understanding of the diverse biological activities of olive-derived compounds, suggesting their potential role in the prevention and management of various chronic conditions.

### 4.2. Mediterranean Diet and Its Health Effects (Green Cluster)

The “Mediterranean diet” (78) stands out as the most significant term in this cluster and is globally renowned for its health benefits. Characterized by a high intake of legumes, vegetables, fish, nuts, fruits, whole grains, seeds, and olive oil, it also includes moderate consumption of alcohol, particularly red wine, while limiting sugary drinks and red meat intake [51]. Olive oil, particularly extra virgin olive oil, is the cornerstone of this diet and is highly valued for its exceptional nutritional quality and bioactive properties [52]. Numerous scientific studies have consistently demonstrated the positive effects of the Mediterranean diet on various aspects of health, from cardiovascular health to cognitive function, as outlined below.

Emerging evidence increasingly suggests that the Mediterranean diet may play a significant role in cancer (36) prevention due to its protective effects, which stem from a reduction in oxidative stress (60) and inflammatory (56) processes within cells. This dietary pattern helps prevent DNA damage, inhibits cell proliferation, and reduces angiogenesis and metastasis [53]. Furthermore, the Mediterranean diet’s high-fiber content and the consumption of plant-based foods provide additional mechanisms for reducing cancer risk, as they promote a healthy gut microbiome, which is increasingly recognized for its role in cancer prevention [54]. Moreover, the health benefits of olive oil extracts, particularly in relation to obesity (21), are linked to their antioxidant properties. These extracts, rich in bioavailable phenolic compounds such as oleuropein, verbascoside, ligstroside, tyrosol, and hydroxytyrosol, not only influence energy metabolism and fat accumulation but also complement the overall protective effects of the Mediterranean diet, further enhancing its potential in reducing cancer risk [55].

In addition to cancer and obesity prevention, the Mediterranean diet and olive oil are recognized for their pivotal role in combating cardiovascular disease (18), which remains the leading non-communicable cause of death in Europe (∼50% of all deaths) [56]. Epidemiological studies have consistently highlighted the protective effects of olive oil consumption against cardiovascular events and all-cause mortality, with a particularly pronounced benefit observed at an intake of up to 20 g/day [57]. These advantages are largely attributed to the high concentration of monounsaturated fatty acids in olive oil, primarily oleic and palmitoleic acids, which contribute to improved lipid profiles, reduced inflammation, and enhanced endothelial function. As a result, incorporating olive oil into the diet, especially in foods enriched with these heart-healthy fats, is strongly recommended to mitigate cardiovascular risk, manage weight, and confer numerous additional health benefits.

The Mediterranean diet and olive oil’s protective effects on cardiovascular health are further enhanced by the powerful polyphenols (174) found in olive. These molecules, renowned for their potent antioxidant (84) properties, are highly effective in reducing free radical formation and scavenging reactive oxygen species, contributing to the prevention of oxidative stress and inflammation [20] Beyond their sensory contributions—such as bitterness, color, and flavor—polyphenols have a broader biological impact. They modulate gene expression involved in various cellular functions, inducing epigenetic changes that promote health [58], and they exhibit a “prebiotic-like” effect, influencing gut microbiota and enhancing the biological activity of plant phenolics through microbial metabolism [59]. These bioactive compounds are credited with the numerous health benefits of olives, including anti-atherogenic, anti-inflammatory, and immunomodulatory effects, which further reinforce olive oil’s role in reducing the risk of cardiovascular diseases and enhancing overall well-being [60].

Although the benefits of the Mediterranean diet on cardiovascular health are well-established, more research is needed to determine the specific role of its components, such as the polyphenols in olive oil, in preventing cardiovascular diseases and cancer. Studies exploring the precise molecular mechanisms by which polyphenols and other dietary components contribute to these preventative effects are still lacking. Additionally, further clinical trials are required to clarify the dose–response relationship between olive oil intake and disease risk, as well as to better understand how factors like individual genetic predispositions, physical activity, and smoking interact with the Mediterranean diet to optimize its health benefits and personalize dietary recommendations

### 4.3. Olive Bioactive Compounds of Interest (Yellow Cluster)

**Figure 3:**
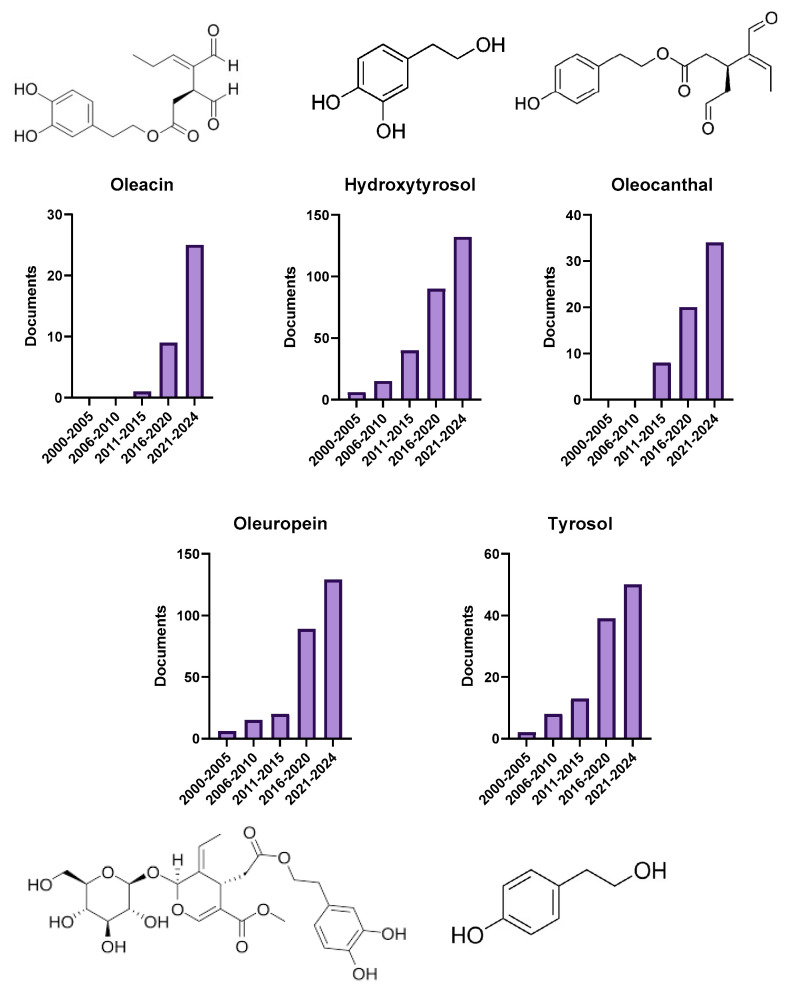
Progression of published documents by lustrum (period 2000–2024).

Olive represents a rich source of unsaturated fatty acids and an array of minor bioactive components, including vitamins, chlorophylls, phytosterols, and polyphenols. However, the health benefits attributed to these compounds are primarily linked to the major secoiridoid (38) derivatives, such as oleuropein (143), oleocanthal (35), and oleacein (18), as well as simple phenols like hydroxytyrosol (158) and tyrosol (39) [61]. According to the co-occurrence data, oleuropein, hydroxytyrosol, and tyrosol have been extensively studied in recent years. Although oleacein and oleocanthal have been less frequently researched, they are gaining increasing attention for their promising biological effects. As illustrated in Figure 3, these compounds demonstrate significant potential in various health-related applications, highlighting the need for further investigation to fully uncover their therapeutic properties, as discussed in the following sections.

Oleuropein, the most prominent phenolic compound and primary secoiridoid constituent found in the olive tree and its leaves, is renowned for its potent antioxidant properties, with its structure (Figure 3), characterized by a hydroxyphenyl group attached to a secoiridoid aglycone, playing a key role in its bioactivity, as the hydroxyl groups in this structure contribute significantly to its antioxidant and anti-inflammatory effects.

Oleuropein has been shown to exert a broad spectrum of beneficial effects, encompassing anti-inflammatory, anticancer, antimicrobial, and neuroprotective actions [62]. Additionally, it has demonstrated efficacy against conditions such as COVID-19 [63] and oxyuriasis [64] and emerging research suggests its potential to mitigate photoaging, underscoring its relevance in skin health and cosmetic applications [65,66]. Future investigations could explore the interaction between oleuropein and the skin microbiome. Understanding how this molecule modulates the composition and functionality of skin-associated microbes may offer valuable insights into its role in maintaining skin health, enhancing its antimicrobial and anti-inflammatory properties, and further supporting its potential in cosmetic and therapeutic formulations.

Various extraction methods, both conventional and modern, have been employed to enhance the yield of oleuropein. These techniques include maceration, Soxhlet extraction, microwave-assisted extraction, ultrasonication, and supercritical fluid extraction, all of which have proven effective in isolating this bioactive compound from olive leaves and other olive derivatives [67]. Despite oleuropein being one of the most studied molecules derived from olives, further research is necessary to improve its absorption and bioavailability. Specifically, understanding the relationship between enhanced bioavailability and the biological efficacy of oleuropein is crucial. Additionally, more clinical trials are required to refine its optimal dosage, explore its mechanisms of action, assess its therapeutic effectiveness, and evaluate its safety in human consumption.

While oleuropein is the most extensively studied phenolic compound in olive oil, its degradation products, such as elenolic acid and hydroxytyrosol, are also crucial contributors to its biological activity and health benefits [68]. This breakdown occurs primarily through enzymatic hydrolysis and oxidation, processes that release these metabolites, thereby enhancing the bioavailability and therapeutic potential of the phenolic compounds. Understanding the specific enzymatic pathways and environmental conditions that facilitate this transformation could play a pivotal role in optimizing the extraction and utilization of these bioactive compounds, not only in food but also in therapeutic applications. The following section highlights the key characteristics of the two prominent metabolites generated from oleuropein’s conversion:

- Hydroxytyrosol, a phenylethanoid compound predominantly found in olive leaves and the oil extracted from the fruit, is renowned for its potent antioxidant properties. Its structure (Figure 3), featuring a catechol moiety with hydroxyl groups at the 3-and 4-positions, contributes significantly to its strong antioxidant activity, as these hydroxyl groups play a crucial role in scavenging free radicals.

Notably, this molecule exhibits an exceptional bioavailability of 99%, enabling its efficient absorption and utilization within the human body [69]. In 2012, the European Food Safety Authority (EFSA) acknowledged hydroxytyrosol’s cardiovascular protective role, as it helps prevent the oxidation of low-density lipoprotein cholesterol by free radicals, thereby maintaining normal levels of high-density lipoprotein and playing a crucial role in the prevention of atherosclerosis [70].

More recently, hydroxytyrosol has been investigated for its potential applications in a variety of fields, revealing its versatility. For example, there is growing interest in its use as a natural pesticide [71], and ongoing research is exploring its potential link to oligozoospermia, suggesting it may influence cilium and microtubule-based movement, thereby potentially improving sperm quality [72]. However, further studies are needed in both areas to comprehensively assess its effectiveness and explore its diverse range of applications.

- Elenolic acid, a significant compound found in mature olives and extra-virgin olive oil, is a product of oleuropein decomposition during the fruit’s maturation process. Recent studies have demonstrated that elenolic acid administration can normalize fasting blood glucose levels and restore glucose tolerance in high-fat diet-induced obese mice, highlighting its potential as both an anti-diabetic and anti-obesity agent [73]. Additionally, research by Salamanca et al. has suggested that olive leaf extract rich in elenolic acid (marketed as Isenolic^®^) could serve as a promising natural alternative to conventional influenza treatments [74]. Beyond these therapeutic effects, the dialdehydic forms of elenolic acid have been identified as the primary antimicrobial agents in olive oil, further underlining the compound’s significant contribution to promoting overall health and well-being [75].

In contrast, tyrosol, another prominent phenolic compound found in olive oil and wine, is recognized for its wide range of biological effects, including antioxidant, anti-inflammatory, and antimicrobial activities. Its structure (Figure 3), characterized by a simple phenolic structure with a hydroxyl group at the 4-position, plays a key role in its antioxidant activity, as it readily donates electrons to free radicals, neutralizing oxidative stress.

This molecule has been identified as a cardioprotective and neuroprotective agent, with emerging potential in anticancer therapy and combating diabetes [76]. Additionally, tyrosol is well-regarded for its beneficial effects on skin health, where its antioxidant, anti-inflammatory, and photoprotective properties contribute to skin regeneration and provide protection against oxidative stress [77,78]. Despite its promising therapeutic benefits, tyrosol’s bioavailability remains relatively low compared to other bioactive compounds in olive oil, which limits its full utilization in clinical settings [79]. Therefore, future research will likely focus on strategies to enhance its bioavailability and maximize its therapeutic potential. One promising approach involves glycosylating tyrosol, which not only reduces its cytotoxicity compared to its unmodified form but also enhances its antioxidant and anti-inflammatory properties [80].

Oleocanthal, despite accounting for only 10% of the polyphenols in olive oil, has garnered significant scientific attention in recent years due to its intriguing biological activities. Its structure (Figure 3), characterized by a phenolic aglycone linked to an aromatic aldehyde group, plays a key role in its anti-inflammatory and antioxidant effects, with the aldehyde group contributing to its ability to inhibit pro-inflammatory enzymes, mimicking the action of ibuprofen.

Recent research has illuminated its pharmacological properties, demonstrating its ability to mitigate inflammation and oxidative stress, while also revealing its potential effects on specific types of cancer, neurodegenerative diseases, and rheumatic conditions [81]. Notably, oleocanthal is responsible for the distinctive pungent sensation associated with extra virgin olive oil, often associated to the throat-irritating effect of ibuprofen. Interestingly, studies have suggested that oleocanthal and ibuprofen may share similar biological mechanisms, prompting the hypothesis that regular oleocanthal consumption could offer protection against a variety of pathological conditions [82,83]. Nevertheless, despite these promising findings, further investigation is required to fully unravel the molecular pathways underlying oleocanthal’s therapeutic effects. More additional clinical trials are also necessary to identify optimal dietary intake levels that would maximize its health benefits while ensuring safety.

Finally, oleacein, the second most abundant secoiridoid in extra virgin olive oil, has garnered scientific interest for its antioxidant and anti-inflammatory properties [84]. Its structure (Figure 3), similar to oleuropein but with a less complex secoiridoid aglycone, contributes to its potent antioxidant activity and ability to modulate inflammation. The presence of a phenolic group and an ester bond plays a significant role in inhibiting pro-inflammatory cytokines, enhancing its therapeutic potential.

Oleacein has also shown potential in *Caenorhabditis elegans*, extending lifespan and enhancing stress resistance [85], and a novel green process has been developed to extract thiocanthal and thiocanthol from oleacein, with potential as anti-inflammatory agents [86]. However, more research is needed to fully elucidate its molecular pathways, therapeutic potential, and effects on human health, especially in clinical settings. Future studies should focus on optimizing production methods and evaluating the safety and efficacy of oleacein-based interventions, as producing large quantities of oleacein remains a challenge, whether through extraction, synthesis, or organic processes.

### 4.4. Other Important Bioactive Compounds of Olive (Blue Cluster)

As highlighted in the preceding sections, the bioactive composition of foods plays a pivotal role in human health, as the compounds present in various foods significantly influence fundamental biological processes. Certain nutrients and bioactive molecules are essential for cellular protection, mitigating oxidative stress, enhancing cellular stability, and bolstering antioxidant defense systems. These compounds are not only crucial in the prevention of chronic diseases but also in fostering overall well-being by preserving cellular integrity against both intrinsic and extrinsic stressors. Moreover, bioactive compounds found in foods, including those that modulate gut health, immune responses, and metabolic pathways, are integral to optimizing health outcomes. As research progresses, there is an increasing emphasis on identifying these molecules, particularly those derived from natural sources. In this regard, the olive, with its abundant array of bioactive compounds, represents a valuable source of such bioactive molecules:

Carotenoids (23) are natural pigments found in a variety of fruits and vegetables, essential for protecting lipid membranes from oxidative damage and acting as precursors to vitamin A, which is vital for functions such as vision, immune support, and skin health. Carotenoids also exhibit a broad spectrum of biological activities, including antioxidant, anti-tumor, anti-diabetic, anti-aging, and anti-inflammatory properties [87]. While the olive tree naturally contains carotenoids, recent research has focused on enhancing the presence of these compounds in olive oil to explore their physical and chemical properties. Studies have demonstrated that the incorporation of carotenoids into olive oil can prevent degradation, extend its shelf life, and improve its nutritional value, all while preserving other bioactive compounds, such as polyphenols [88]. Furthermore, Garrido et al. [89] investigated the health benefits of virgin olive oil enriched with lycopene, a potent carotenoid, and demonstrated its potential to enhance the antioxidant effect of olive oil. Their findings suggest that lycopene-enriched olive oil may help combat diseases such as coronary heart disease and certain cancers, where oxidative stress plays a crucial role.

Another crucial group of bioactive compounds are fatty acids (47), which play a vital role in human health, especially in the regulation of lipid metabolism, hormone production, and the prevention of chronic diseases. Oleic acid (22), the dominant fatty acid in olive oil (comprising 63–79% of the fatty acid profile in extra virgin olive oil), is widely recognized for its cardiovascular benefits, including reducing harmful LDL cholesterol levels while increasing HDL cholesterol, thus supporting a healthier cardiovascular system [90,91]. In addition to its effects on lipid regulation, oleic acid enhances endothelial function by protecting against oxidative stress and postprandial vascular damage, alleviates hypertension through improved nitric oxide bioavailability, and reduces systemic inflammation [90]. Oleic acid also stabilizes atherosclerotic plaques [91] and mitigates insulin resistance [92], highlighting its broader impact on cardiovascular and metabolic health.

Finally, other essential bioactive compounds in olive oil, such as squalene (26) and tocopherols (33), contribute significantly to the nutritional value of the Mediterranean diet. Squalene, a triterpene with antioxidant, anti-inflammatory, anticancer, and anti-atherosclerotic properties, is particularly noted for its liver-protective potential and its role in disease prevention [93,94]. Recent research has focused on extracting squalene from olive oil by-products, such as olive pomace and wastewater [95], enhancing the sustainability of olive oil production. By utilizing these residues, the olive oil industry can provide a renewable source of squalene, benefiting both the pharmaceutical and cosmetic sectors. Meanwhile, tocopherols, including α-, β-, and γ-tocopherol, are potent antioxidants that neutralize free radicals and reactive oxygen species, playing a vital role in preventing age-related diseases, cardiovascular conditions, and Alzheimer’s disease [96,97,98]. Despite the well-documented health benefits of tocopherols, further research is needed to fully understand their molecular mechanisms, clinical effectiveness, and to determine optimal dosages and long-term effects within the Mediterranean diet.

### 4.5. The Power of Olive’s Terpenes (Purple Cluster)

In parallel with the research on the previously mentioned compounds, studies on terpenoids have gained significant momentum due to their wide-ranging health benefits, including anti-inflammatory, antioxidant, and anticancer activities [99]. The main triterpene from olive leaves is oleanolic acid (32), followed by significant concentrations of maslinic acid (25) and minor amounts of ursolic acid, erythrodiol, and uvaol. As a result, various efforts have been made to extract these compounds using sustainable, green methods. For instance, Agatonovic-Kustrin et al. [100] successfully extracted maslinic and oleanolic acids from olive flower extract using high-performance thin-layer chromatography. Similarly, Yang et al. (2024) [101] employed high-speed shear off-line coupled with high-speed countercurrent chromatography to extract these compounds from olive pomace. Additionally, microwave-assisted extraction has emerged as an efficient technique for obtaining maslinic and oleanolic acids from wet olive pomace, offering a high yield and minimal processing [102,103].

**Figure 4:**
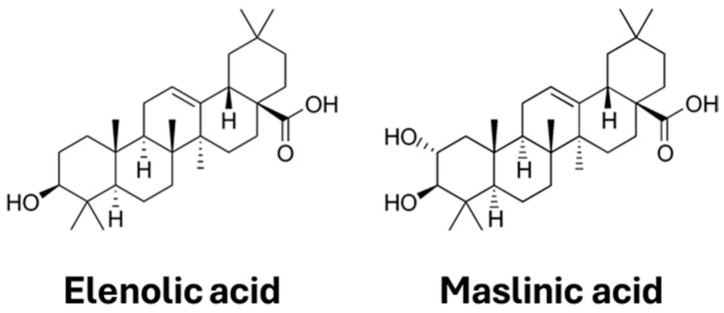
Structure of the two main terpenes in olive.

More specifically, maslinic acid is a pentacyclic triterpene acid nutraceutical found in various plants, recognized for its safety and diverse biological activities, including anti-inflammatory, antioxidant, anti-tumor, hypoglycemic, and neuroprotective effects [104]. Its structure (Figure 4), characterized by a pentacyclic triterpene framework with a carboxylic acid group, plays a key role in its bioactivity. This structural feature is crucial for its ability to modulate various signaling pathways, contributing to its antioxidant and anti-inflammatory effects.

Recent research has drawn attention to its potential to modulate the gut microbiota, a promising avenue of investigation. This modulation could decrease the abundance of harmful bacteria while promoting beneficial ones, potentially alleviating gastrointestinal disorders, such as alcohol-induced liver damage, via the gut–liver axis [105]. Additionally, maslinic acid has been studied for its ability to inhibit the SARS-CoV-2 main protease with promising results [106] and for mitigating denervation-induced loss of skeletal muscle mass and strength [107,108]. However, as this research is still in its preliminary stages, further studies are needed to fully explore its therapeutic potential.

Oleanolic acid, a naturally occurring pentacyclic triterpenoid, has garnered significant attention in recent years due to its promising therapeutic potential, as numerous studies have highlighted its beneficial effects on a range of diseases and conditions [109]. Its structure (Figure 4), characterized by a pentacyclic triterpenoid framework with a hydroxyl group at position 3, plays a key role in its bioactivity. This structural feature is important for its ability to interact with cellular receptors and modulate various signaling pathways, contributing to its anti-inflammatory and hepatoprotective properties.

For instance, Sureda et al. suggested that oleanolic acid may help lower blood pressure, positioning it as a promising adjunct to conventional hypertension treatment [110]. This triterpenoid has also demonstrated hepatoprotective properties by reducing liver damage and promoting functional recovery in both in vitro and in vivo models [111,112]. Moreover, recent research indicates that oleanolic acid alleviates colitis in mice by modulating inflammation-related pathways and enhancing the body’s natural defense mechanisms, underscoring the need for further exploration of its therapeutic applications [113]. Future studies should also examine its interaction with the gut microbiota, as this may be key to its anti-inflammatory effects and overall health benefits.

## 5. Conclusions and Future Prospects

Our bibliometric analysis highlights a marked growth in olive bioactivity research since 2000, driven by the growing interest in the health-promoting potential of compounds such as polyphenols, carotenoids, and secoiridoids. These bioactive compounds have shown considerable promise in the prevention and management of chronic conditions, including cardiovascular, metabolic, and neurodegenerative diseases, primarily through the modulation of inflammation, oxidative stress, and the gut microbiota.

Recent analytical advancements have improved our understanding of their bioavailability, metabolism, and synergistic effects, opening new avenues for applications in functional foods, personalized nutrition, and preventative health strategies. In parallel, green extraction methods and nanotechnology are emerging as promising tools to optimize yield, enhance compound stability, and improve sustainability.

Despite this progress, significant knowledge gaps remain. Well-designed, controlled clinical trials are urgently needed to establish effective dosages and confirm therapeutic benefits. Moreover, research remains geographically biased toward Mediterranean cultivars, overlooking the potential of diverse olive varieties from under-represented regions such as North Africa, the Middle East, and Asia. Expanding the phytochemical profiling of these cultivars may lead to the identification of novel compounds with unique bioactivities. In addition, compounds such as oleuropein, elenolic acid, and newly identified phenolics warrant further exploration using advanced omics technologies to better understand their mechanisms of action.

The integration of interdisciplinary research, including nutrition science, pharmacology, microbiology, and computational biology, will be key in translating current findings into practical applications. The valorization of olive by-products through circular economy strategies and biorefinery approaches also holds great prospects for enhancing sustainability across industries such as food, cosmetics, nutraceuticals, and pharmaceuticals.

Finally, while bibliometric analysis provides valuable insight into research trends, it should be complemented by systematic reviews, meta-analyses, and alternative metrics (altmetrics) to help mitigate publication and citation biases. Combining these approaches will enhance the accuracy and impact of future evaluations in this rapidly evolving field.

## Data Availability

The original contributions presented in the study are included in the article, further inquiries can be directed to the corresponding author.
